# Evaluation of Hazel-Derived Particleboard as a Substitute for Conventional Wood-Based Composites

**DOI:** 10.3390/ma18163773

**Published:** 2025-08-12

**Authors:** Marta Wronka, Damian Wojnicz, Anita Wronka, Grzegorz Kowaluk

**Affiliations:** 1 Faculty of Human Nutrition, Warsaw University of Life Sciences−SGGW, Nowoursynowska St. 159, 02-776 Warsaw, Poland; s223022@sggw.edu.pl (M.W.); s223012@sggw.edu.pl (D.W.); 2 Institute of Wood Science and Furniture, Warsaw University of Life Sciences−SGGW, Nowoursynowska St. 159, 02-776 Warsaw, Poland; anita_wronka@sggw.edu.pl

**Keywords:** biomass utilization, wood alternatives, hazel wood, single-layer particleboard

## Abstract

This study investigated the potential of hazelnut wood (*Corylus avellana* L.) as an alternative raw material in the production of single-layer structural particleboards. Boards with a target density of 700 kg m^−3^ and thickness of 13 mm were manufactured using varying substitution levels (5%, 10%, 25%, 50% and 100%) of hazel wood particles relative to industrial pine (*Pinus sylvestris* L.) particles. Phenol-formaldehyde (PF) resin was used as the adhesive at a 15% resination rate. Mechanical and physical properties, including modulus of rupture (MOR), modulus of elasticity (MOE), internal bond (IB), screw withdrawal resistance (SWR), water absorption (WA), and thickness swelling (TS), were evaluated according to relevant European standards. Density profiles (DP) were also assessed. The results showed that while higher hazel content reduced bending strength (from 23.3 N mm^−2^ for reference to 18.7 N mm^−2^ for 100% hazel wood board) and stiffness (from 3515 N mm^−2^ for reference to 2520 N mm^−2^ for 100% hazel wood board), most boards met standard mechanical requirements of EN 312 for P3 and P5 boards. Notably, IB strength improved significantly at higher hazel content, with the 100% variant (2.07 N mm^−2^) exceeding the reference board (1.57 N mm^−2^). Screw withdrawal resistance also increased with hazel wood addition (from 235 N mm^−1^ for reference to 262 N mm^−1^ for 100% hazel wood board), linked to its higher density. However, water resistance and dimensional stability worsened with increasing hazel content, particularly in bark-containing particles, leading to excessive thickness swelling after prolonged water exposure. Thickness swelling after 24 h of soaking rose from 16.36% for the reference board to 20.13% for the 100% hazel wood board. Density profiles revealed a more uniform internal structure in boards with higher hazel content. Overall, hazelnut wood shows promise as a partial substitute for pine in particleboard production, especially at moderate substitution levels, though limitations in moisture resistance must be addressed for broader industrial application.

## 1. Introduction

The world’s production of wood-based panels reaches nearly 358 million cubic metres per year [1]. Poland has long been among the leading countries in producing and importing these materials. In 2022, Poland produced over 11 million cubic metres of wood-based panels, ranking fourth in Europe after Germany, Turkey, and Russia. The production of sawnwood reached approximately 7.6 million cubic metres, also placing Poland fourth in the UNECE region. In addition, Poland was among the leading European producers of wood pellets, with output exceeding 1.5 million tonnes [2].

This increase was largely driven by the rapid growth of the furniture industry, which relies heavily on particleboard. Import figures confirm this trend, as Poland consistently ranks among the top importers, following only Germany and the United States [3].

Particleboard is a key wood-based material, produced from wood particles bonded with synthetic resin and compressed under high pressure and temperature. In recent years, its production and global demand have been steadily increasing. It is an economical and ecological way to utilize wood waste, underutilized and/or low-quality wood and is widely used in construction and furniture manufacturing.

Although research has been ongoing into the use of alternative raw materials such as agricultural biomass or fast-growing tree species, wood remains the primary raw material. It comes mainly from by-products of mechanical processing [4]. There is also a growing use of post-consumer wood, which comes from windows, doors, furniture, and packaging. This raw material often contains admixtures of glass, metal, plastics, residues of adhesives, paints, and preservatives. Proper segregation, cleaning, and shredding make it possible to turn this raw material into a valuable resource for producing wood-based panels [5]. There is growing demand from industries for lignocellulosic raw materials such as particleboard, fiberboard, and other wood-based composites manufacturing, where thermal energy generation, and pulp and paper production is intensifying competition. To prevent resource shortages, it is essential to promote efficient utilization and sustainable management of these materials [6].

By-products such as branches, bark, and pinecones are increasingly being used in the production of particleboards. An example includes Scots pine (*Pinus sylvestris* L.) and spruce wood, both of which are widely used in the furniture industry, construction, and the production of wood-based materials. In the study, material obtained from Scots pine branches was used; it originated from post-logging residues left in the forest after harvesting [7]. An attempt was made to use spruce branches (*Picea abies*) as a wood material. Branch wood has similar mechanical properties to trunk wood but higher density and more compression wood, affecting its stability. Panels made from branches require special processing techniques, including strong adhesives and uniform mats, to prevent cracking. Branches can be a valuable raw material, but their processing requires adapted technology [8]. So far, many alternative lignocellulosic materials have been tested, such as kenaf [9], flax, hemp [10], miscanthus, rapeseed stalks, and cereal residues. Some of these are intentionally cultivated, while others are agricultural by-products. Their availability depends on seasonality and local climatic and soil conditions, and the effects of long-term storage on their properties are poorly understood. Due to technological limitations, such as lower strength and higher mineral content, only a few of these materials have been industrially adopted. They often require more adhesive and adjusted pressing parameters, which affect production economics. Agro-food industry residues like beet pulp and nutshells have been less studied despite their potential as valuable lignocellulose sources [11].

Numerous studies confirm that alternative raw materials can successfully serve as at least a partial substitute for conventional wood in particleboard production. These materials sometimes improve all or some of the tested parameters; however, everything also depends on the intended use of the boards. The fact that some of them do not meet certain standards does not mean they are useless. Examples include boards made from plum and apple wood. Research has shown that this raw material, obtained from the annual pruning of fruit trees, can be used as an additive in particleboard production. This is a positive development, as wood is usually disposed of by burning [12,13]. With this solution, carbon dioxide is successfully sequestered from the atmosphere. Incorporating lignocellulosic materials into particleboard production helps alleviate wood resource shortages and transforms agricultural waste into valuable raw materials. This approach not only reduces waste generation but also promotes the efficient use of available biomass.

Moreover, the use of lignocellulosic biomass in the manufacturing process can significantly reduce harmful emissions. For instance, replacing traditional adhesives with liquefied lignocellulosic biomass and nanocellulose-based formulations has been shown to lower emissions of formaldehyde and volatile organic compounds (VOCs) [14,15,16]. Additionally, barley husks, oat husks, and wheat bran, due to their hemicellulose and lignin content being comparable to that of industrial wood chips, are suitable raw materials for manufacturing non-load-bearing particleboards [11]. Elephant dung, combined with bamboo layers, has been utilized to create particleboards with improved physical and mechanical properties [17]. Willow and poplar species contribute to panels with superior mechanical performance compared to reference boards, making them suitable for use in the construction and furniture industries [18]. Additionally, cup-plant, sunflower, and topinambour have been applied in particleboard manufacturing, yielding boards with satisfactory properties, although slightly lower than those of conventional spruce particleboards [19]. Another promising material is sugarcane bagasse, which is abundantly available in Brazil and has been successfully used for particleboards, particularly in flooring applications [20]. Although lignocellulosic raw materials can sometimes reduce the mechanical properties of composites, various modification techniques, such as the use of ionic liquids, help to neutralize these negative effects, enhancing both thermal stability and mechanical performance, and making the materials more suitable for industrial applications [21,22]. Panels produced from hemp shives bonded with melamine-formaldehyde adhesive demonstrate greater staple withdrawal resistance than conventional industrial particleboards [23].

Additionally, mechanical properties such as the modulus of elasticity (MOE) are strongly influenced by particle geometry, board structure, and processing parameters. The size and distribution of particles, particularly in the core layer, have been shown to play a crucial role. Boards with normal-sized core particles provide better MOE values than those with significantly thinner or thicker particles [24]. Moreover, adjusting the ratio of fine particles in the face layer can improve mechanical performance; increasing their share up to 30% enhances MOE, while further increases bring no additional benefit [25].

*Corylus avellana* L. (European hazel) is a species widely distributed across Europe and western Asia, from central Scandinavia to Cyprus, and from Ireland to the Urals and the southern Caspian coast. In Poland, large-scale hazelnut cultivation began more recently, gaining traction in the early 2000s. As of the most recent data, annual production in 2017, for example, reached about 4600 tonnes and by 2022 grew to nearly 9500 tonnes [26,27]. Other important producers include the United States, Azerbaijan, Georgia, China, Iran, Spain, France, Kyrgyzstan, Poland, and Croatia [26]. Hazel wood is strong, hard, and features a uniform grain. It is characterized by diffuse porosity, vessels of similar size throughout the annual growth ring, aggregated rays, and spirally thickened vessel elements. It also has scalariform perforation plates composed of 5–10 bars. The average wood density is 0.517 g cm^−3^. As the plant ages, the bark of its shoots hardens, forming a durable structure, which enhances its potential as a timber resource [28].

The aim of the research was to investigate how the inclusion of hazel wood affects the basic strength and physical parameters of single-layer particleboards. This study extends previous research on alternative lignocellulosic raw materials by demonstrating the feasibility of producing single-layer particleboards from hazel (*Corylus avellana* L.) wood particles, a largely underutilized wood resource. Prior work has predominantly focused on agricultural residues or conventional hardwoods, whereas the present work addresses the processing adaptability and performance potential of a non-traditional, regionally abundant species. Hazel wood is particularly attractive due to its favorable density, relatively uniform particle geometry, and widespread availability as a byproduct of orchard management. The findings provide novel data on board properties and their correlation with the characteristics of hazel wood particles, thereby broadening the raw material base for particleboard manufacturing and supporting sustainable resource diversification within the wood-based panel sector.

## 2. Materials and Methods

### 2.1. Materials, Preparation of Panels, and Characterization of the Elaborated Panels

A single-layer structural particleboard with a nominal density of 700 kg m^−3^ and a thickness of 13 mm was manufactured for this study. The panels measured 320 mm × 320 mm and were composed primarily of industrial wood particles derived from *Pinus sylvestris* L. As many as 3 repetitions (panels) of the dimensions mentioned above have been prepared for every tested variant. Hazelnut (*Corylus avellana* L.) wood particles were used as an alternative lignocellulosic raw material, introduced at substitution levels of 5, 10, 25, 50, and 100 wt% relative to the total dry particle mass. Hazel (*Corylus avellana* L.) rods with diameters ranging from 20 to 50 mm were collected in Masovian Voivodeship, Poland, during the spring of 2025. The initial moisture content (MC) of the hazel wood and bark was measured at 86.3% and 75.4%, respectively, with a bark mass ratio of 9.5% in the wet state. The rods were cut into chips measuring 40–50 mm in length and dried in a laboratory kiln (THERMO SCIENTIFIC Oven Series 9000, Thermo Fisher Scientific, Waltham, MA, USA) at 70 °C. The moisture content of the hazel wood chips before particle formation was about 8.2% ± 2.1% and 9.6% ± 2.7%, respectively, for in-bark and debarked chips. Following drying, the material was processed into finer particles using a laboratory hammer mill (prototype; Research and Development Centre for Wood-Based Panels Sp. z o. o., Czarna Woda, Poland) fitted with three knives, two counter-knives, and a 6 mm × 12 mm mesh screen. The resulting particles were separated on 8 mm and 2 mm sieves to collect particles smaller than 8 mm but larger than 2 mm. For reference, 8/2 mm fraction industrial wood particles—consisting of over 95% Scots pine (*Pinus sylvestris* L.)—were obtained from a commercial particleboard manufacturing facility in Poland. A control board (0%) was produced under the same conditions using only pine particles. The substitution levels have been tested in many previous studies [18,29,30,31,32] and represent discrete, practical experimental levels often chosen in material research to (1) cover the full range from no substitution (0%) to full substitution (100%), (2) include low, medium, and high replacement ratios to observe both incremental and threshold effects, and (3) allow for nonlinear property changes that are common in composite materials. Part of the hazel wood chips were debarked before particle production, and these particles have been used to produce 100 DB panels. The remaining panels have been produced from the in-bark hazel wood. The adhesive system consisted of phenol-formaldehyde (PF) resin (with a dry mass content of 49%, estimated in accordance with EN 827 [33]; viscosity (at 25 °C) about 330 mPa s; pH 11.8; curing time (at 100 °C) 128 s; specific gravity (at 20 °C) around 1.22 g cm^−3^), which was supplied by the Research and Development Centre for Wood-Based Panels Sp. z o. o., Czarna Woda, Poland and applied at a resination rate of 15% based on the dry mass of the particles. No wax or other additives were used. The configuration of the tested panels is displayed in Table 1.

All manually formed mats were hot-pressed using an AKE press (AKE, Mariannelund, Sweden) at a temperature of 200 °C, under a peak specific pressure of 2.5 MPa, and a pressing time of 20 s·mm^−^^1^. Such pressing parameters have been chosen based on the previous research on particleboards with alternative raw material particles [18,29,30,31,32]. The pressure was applied during pressing as follows: 0–50% pressing time—max specific pressure; 50–70% pressing time—2/3 max specific pressure; 70–90% pressing time—1/3 max specific pressure; 90–100% pressing time—pressure reduction to 0 and press opening. Following pressing, panels were conditioned for seven days at 20 °C and 65% relative humidity and sanded to the target thickness prior to testing. This study evaluated the mechanical and physical properties of the particleboards, including the modulus of rupture (MOR) and modulus of elasticity (MOE), following the relevant European standards where applicable [34]; the internal bond (IB) strength was measured in accordance with the EN 319 standard [35]. The screw withdrawal resistance (SWR) [36], water absorption (WA), and thickness swelling (TS) were measured after 2 and 24 h of immersion in water [37]. All mechanical properties were evaluated using a computer-controlled universal testing machine (Research and Development Centre for Wood-Based Panels Sp. z o. o., Czarna Woda, Poland). Each mechanical and physical test involved at least eight samples per panel type. To avoid the leading density differences’ influence on the tested parameters, the samples of every tested variant were selected to represent similar densities. For the density profile (DP) measurements, specimens measuring 50 mm × 50 mm were assessed with a Grecon DA-X device (Fagus-GreCon Greten GmbH & Co. KG, Alfeld/Hannover, Germany) using direct X-ray densitometry, scanning the panel thickness in 0.02 mm increments. A representative density profile was selected after analyzing three samples of each variant for further examination. Where applicable, the results were compared to the European standards [38].

### 2.2. Statistical Analyses

Statistical analyses were performed using analysis of variance (ANOVA) and *t*-tests (α = 0.05) to determine significant differences between the examined factors and their levels, with calculations carried out in IBM SPSS Statistics Base (IBM SPSS 20, Armonk, NY, USA). When significant effects were detected, Duncan’s post hoc test was applied to compare the means. The letters “a”, “b”, “c”, etc., in the plots indicate statistically homogenous groups. Additionally, for clarity, mean values of the tested properties along with standard deviations (displayed as error bars) are presented in the corresponding graphs.

## 3. Results and Discussion

### 3.1. Determination of Modulus of Rupture and Modulus of Elasticity in Bending and of Bending Strength

**Figure 1:**
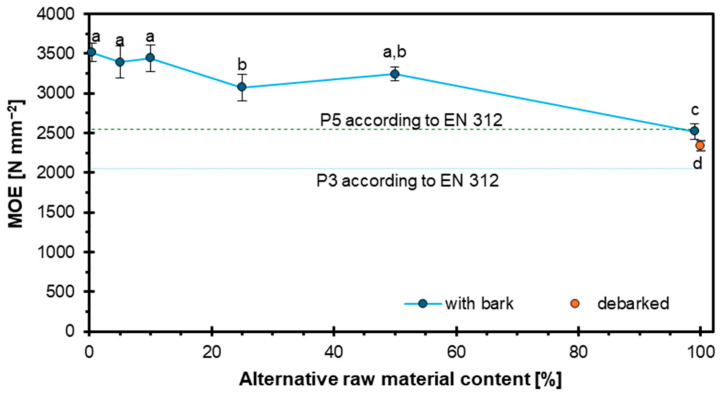
Influence of hazel wood addition on MOE of particleboards.

Figure 1 presents the effect of hazel wood addition on the modulus of elasticity (MOE) of the particleboards. The highest MOE values were recorded for the 0%, 5%, and 10% hazel wood variants, amounting to 3515 N mm^−2^, 3395 N mm^−2^, and 3446 N mm^−2^, respectively. Intermediate values were observed for the 25% and 50% variants, at 3078 N mm^−2^ and 3242 N mm^−2^. The lowest MOE value was obtained for the board produced entirely from the alternative raw material. Considering the standards for P3 and P5 boards, only the variant made entirely from alternative raw material failed to meet the requirements, with a difference of 8 N mm^−2^. The result obtained is satisfactory compared to other alternative raw materials, as the standards were met in most variants. This indicates that hazel wood can largely serve as a substitute. In many cases, raw material substitution in particleboard technology leads to a reduction in the MOE, as observed in boards made with bagasse. While some studies report that particleboards produced from bagasse exhibited superior mechanical properties, including MOE, compared to those made from poplar and mixed hardwoods [39], other research indicates that alternative materials such as sorghum bagasse generally result in lower strength properties, including MOE [40]. Similarly, the incorporation of seaweed in particleboard formulations has been shown to reduce MOE values as its content increases.

In terms of physical parameters, board density is directly correlated with MOE—higher-density boards generally exhibit greater stiffness, while lower-density variants tend to show reduced values [41,42]. Pressing conditions are equally important; longer press times and higher temperatures (e.g., 180 °C) contribute to improved MOE by promoting stronger bonding between particles. Additionally, adhesive content significantly affects MOE: particleboards with higher resination (e.g., 11%) demonstrate enhanced bonding quality and, consequently, increased stiffness [43].

**Figure 2:**
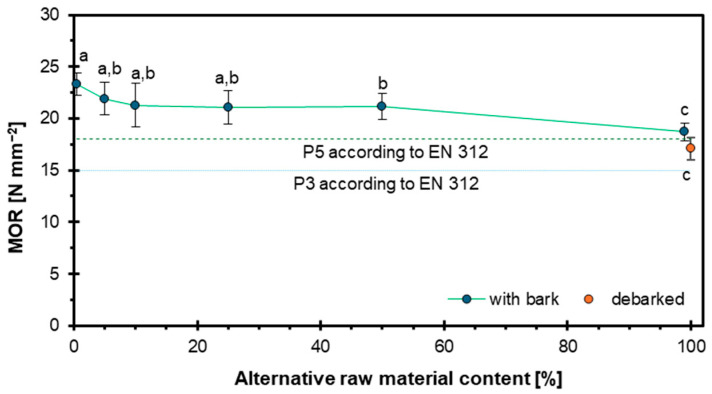
Influence of hazel wood addition on MOR of particleboards.

In Figure 2, the effect of the addition of hazel wood particles on flexural strength is presented. The values recorded for the respective variants are as follows: 23.3 N mm^−2^, 21.9 N mm^−2^, 21.3 N mm^−2^, 21.0 N mm^−2^, 21.2 N mm^−2^, 18.7 N mm^−2^, and 17.1 N mm^−2^. Thus, it can be observed that with the increasing share of the alternative raw material, a decrease in flexural strength was noted. Nevertheless, the produced boards still meet the requirements of European standards, which should be considered an advantage, as in many cases the use of alternative raw materials leads to a significant deterioration of strength properties. The observed decrease in flexural strength with increasing content of hazel wood particles can be attributed to the anatomical characteristics of hazel wood. Specifically, hazel wood formed at the top of junctions tends to exhibit more tortuous grain patterns. While such grain irregularity may enhance radial and tangential tensile strength, it also introduces discontinuities and stress concentrations that reduce the material’s ability to withstand bending forces. The complexity of the grain structure likely disrupts the uniform distribution of stress under flexural load, leading to diminished bending strength in boards with higher hazel content [44]. The presence of knots in hazel wood contributes to reduced flexural strength, as larger knots and their proximity to the loading point increase the risk of fracture by disrupting stress distribution and weakening the structure [45].

### 3.2. Internal Bond

**Figure 3:**
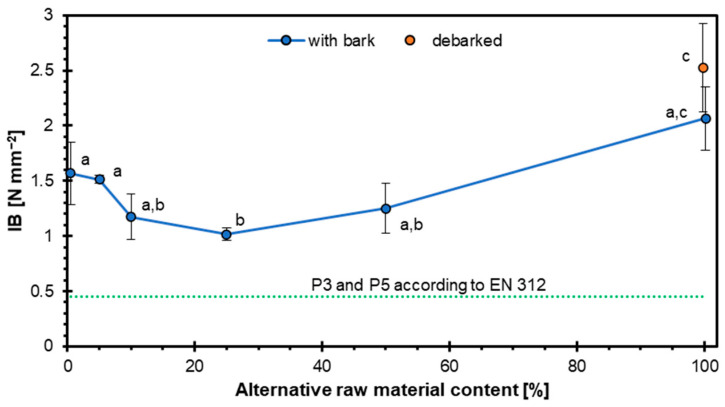
Influence of hazel wood addition on IB of particleboards.

The results of the IB test are presented in Figure 3. The recorded values for successive variants were as follows: 1.57 N mm^−2^, 1.52 N mm^−2^, 1.17 N mm^−2^, 1.02 N mm^−2^, 1.25 N mm^−2^, 2.07 N mm^−2^, and 2.52 N mm^−2^. It was observed that the IB strength initially decreased with the increasing proportion of alternative raw material, reaching a minimum at approximately 30–40% content. Beyond this point, however, a steady increase in IB values was noted, ultimately exceeding even the reference variant (0% alternative content). The drop in IB strength when substituting 5–25% of pine particles with hazel wood is mainly attributed to hybrid effects arising from differences in wood mechanics and surface properties. Hazel wood introduces localized weak interfaces within the otherwise optimized pine-based structure. At low substitution (5–25%), hazel particles are dispersed within a pine-dominated matrix, causing localized stiffness variations and weak bonding zones. Under IB testing, these weaker hazel interfaces act as stress concentrators, leading to early crack initiation. At higher substitution (e.g., 100% hazel), the structure is homogeneous again, and stress distribution is more uniform, leading to improved IB. Such hybrid defects are well documented in the wood-based composite literature, where mixed raw materials often reduce bonding efficiency until pressing parameters are re-optimized or single-source raw material is used [46,47].

All tested variants met the minimum IB requirements specified in the EN 312 standard [38] for P3 and P5 boards (0.5 N mm^−2^), as indicated by the dashed line on the graph. Notably, the sample containing 100% alternative raw material exhibited the highest IB value (2.52 N mm^−2^), more than 60% higher than that of the reference variant.

These findings indicate that while a moderate addition of alternative raw material may initially reduce internal bond strength, higher substitution levels can lead to a substantial improvement in this property. This nonlinear trend suggests a complex interaction between material composition and bonding mechanisms. Importantly, the maintenance of compliance with European standards across all formulations highlights the potential of hazel wood particles as a viable alternative raw material in board production.

A review of the literature confirms that, in certain cases, the substitution of conventional raw materials can result in improved IB strength. Such an effect has been observed, for example, in particleboards manufactured with willow wood [48]. An additional explanation for these results may lie in the composition of the particleboard, including the type of wood particles used, which significantly influences IB strength. Hazel wood particles, due to their higher density of solid hazel wood [49] and thus higher heat transfer dynamics during pressing [50], may exhibit better bonding properties and higher compatibility with adhesives, leading to increased IB strength compared to pine wood particles [51]. As a further explanation for the observed results, attention should also be given to the density and structural characteristics of the particleboards. These parameters have a significant impact on IB performance. Boards produced with hazel wood particles may demonstrate a more consistent density distribution and improved mechanical integrity, resulting from both the inherent properties of hazel wood and the specific processing conditions applied [52].

### 3.3. Water Absorption and Thickness Swelling

**Figure 4:**
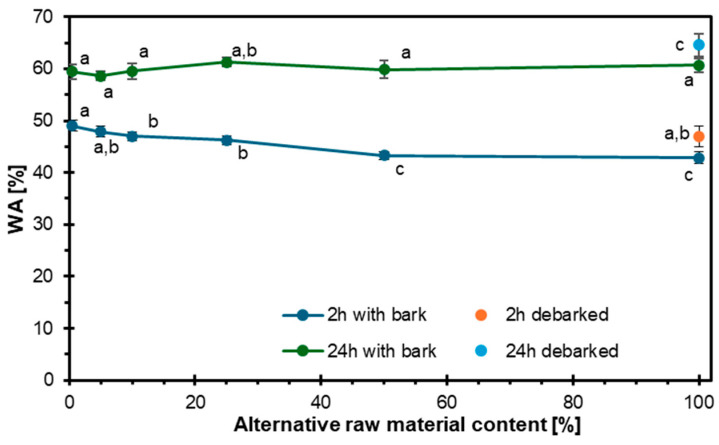
Influence of hazel wood addition on water absorption of particleboards.

Figure 4 presents the water absorption results after 2 and 24 h for particleboards made from debarked and non-debarked hazel wood particles. During the first two hours, it is evident that a higher proportion of alternative raw material corresponds to lower water absorption. When comparing the variants based on the debarking of the raw material, the non-debarked variant exhibits a higher susceptibility to water absorption. Over the 24 h period, water absorption increases compared to the 2 h measurements, and the average absorption in the variant composed entirely of hazel wood exceeds that of the reference variant.

The lower water absorption of particleboard variants with a higher proportion of alternative materials during the first two hours can be attributed to differences in porosity structure and the content of hydrophobic extracts in the materials used, as porosity significantly influences water absorption, where materials with higher porosity absorb more water due to the increased surface area and void spaces [53,54], and denser materials with fewer pores exhibit lower water absorption [55,56]. Moreover, the dual effect of micro- and macropores, where micropores trap water, slowing its movement, and macropores allow for quicker but saturable water uptake, explains the initially lower absorption in materials with a more complex pore structure [57,58]. Additionally, the hydrophobicity of materials plays a crucial role, since hydrophobic extracts repel water and reduce absorption [59,60,61], and softwoods, which typically contain more hydrophobic compounds such as resin acids and phenolics, have lower water absorption compared to hardwoods [61,62], whereas the lower content of hydrophobic extracts in hazel wood relative to softwoods may lead to increased water absorption [59,61]. The observed lower water absorption in particleboards containing higher amounts of hazel wood during the first two hours results from a combination of a more complex pore structure and a lower content of hydrophobic extracts compared to traditional softwoods, contributing to slower initial water uptake and potentially improving short-term moisture resistance.

**Figure 5:**
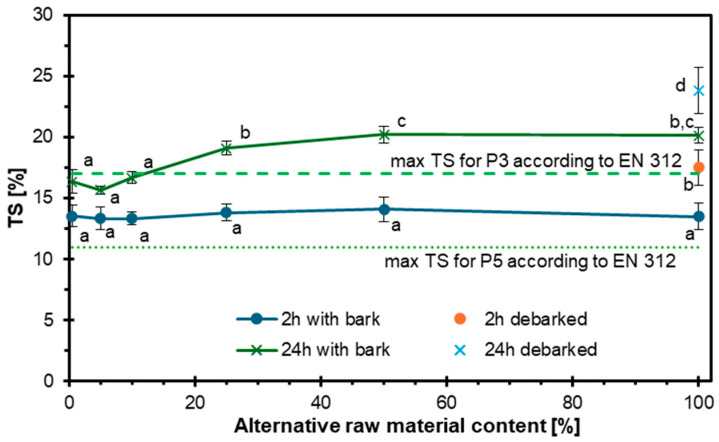
Influence of hazel wood addition on the thickness swelling of particleboards.

Figure 5 presents the TS of single-layer particleboards after 2 and 24 h of water immersion, depending on the content of hazel wood used as an alternative raw material (ranging from 0.5% to 100%). The tested variants included both barked and debarked hazel wood. After 2 h, TS remained relatively low and stable across all samples (approximately 13–15%), regardless of bark presence. However, after 24 h, TS increased significantly, particularly in samples containing bark, and this increase was clearly correlated with the rising proportion of hazel wood. The highest TS values after 24 h were observed for boards made entirely from hazel wood, especially those with bark, exceeding the maximum allowable values specified in EN 312 [38] for P5 (14%) and P3 (17%) particleboard types. Statistical differences between groups are indicated by different letters above the data points. Overall, the addition of hazel wood, particularly with bark, negatively affected the water resistance of the boards, especially over prolonged exposure times. While boards with a low hazel content (up to 10%) remained within the standard limits, higher substitution levels led to a loss of dimensional stability and failed to meet EN 312 [38] requirements. There may be several reasons for this phenomenon, including the presence of wood bark (which refers to the panels with in-bark particles), which contains a higher amount of extractives, hemicelluloses, and capillary pores than wood without bark, increasing its susceptibility to water absorption and swelling [61,63,64].

### 3.4. Screw Withdrawal Resistance

**Figure 6:**
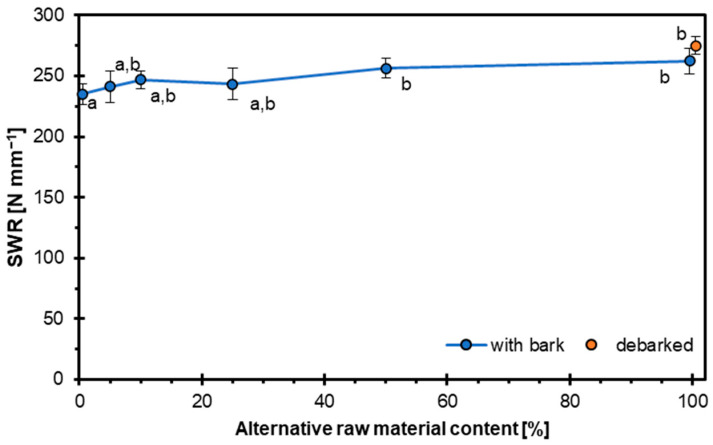
Influence of hazel wood addition on SWR of particleboards.

The results of screw withdrawal resistance are presented in Figure 6. The average values for the respective variants are as follows: 235 N mm^−1^, 241 N mm^−1^, 247 N mm^−1^, 243 N mm^−1^, 256 N mm^−1^, and 262 N mm^−1^. It can be observed that with an increasing proportion of alternative raw material, the SWR increases, which is a favorable phenomenon. This relationship can be explained by the density of hazel wood, which is higher than that of pine wood [65,66,67]. Wood’s capacity to hold fasteners, such as screws, is strongly affected by its density. Denser wood offers greater resistance to screw withdrawal and enhances the load-bearing capacity of fasteners [68,69,70]. This occurs because higher-density wood provides a larger amount of solid material for the screw threads to engage with, thereby reducing the risk of fastener pull-out or failure [71,72]. In addition to the properties of hazel wood as an alternative raw material, screw withdrawal resistance is also influenced by the characteristics of the particleboard itself, such as its target density, as well as the type and amount of adhesive used. Pressing parameters also play a crucial role, as they determine the compaction level of the board, directly affecting its internal structure and density distribution. These factors collectively influence the mechanical strength and fastening capacity of the finished particleboard [73,74].

### 3.5. Bulk Density

**Figure 7:**
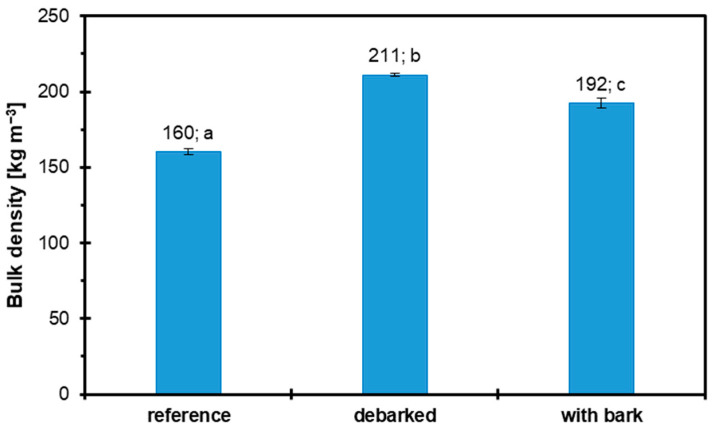
Bulk density of reference, debarked, and bark-containing particles.

The bulk density of industrial wood particles, debarked hazel wood, and non-debarked hazel wood is presented in Figure 7. The lowest bulk density was recorded for industrial wood particles at 160 kg m^−3^, which is typical for this type of raw material, characterized by a large number of void spaces between irregularly arranged and often larger particles, and the results are consistent with those reported in other studies [30]. In contrast, the highest value was observed for debarked hazel wood at 211 kg m^−3^. Intermediate values were noted for the variant containing hazel wood with bark, amounting to 192 kg m^−3^. Bark generally has a lower density than wood and a larger volume, which results in wood with bark having a lower bulk density, while debarked wood has a higher bulk density [75].

### 3.6. Density Profiles

**Figure 8:**
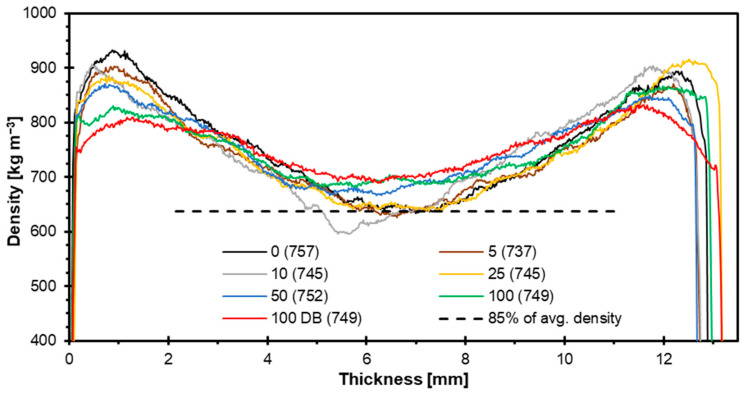
Density profiles of tested particleboards.

The density profile of the individual variants is presented in Figure 8. All profiles exhibit a characteristic U-shape, with higher density values in the surface layers and a distinct reduction in the core zone, which is typical for hot-pressed particleboards. The reference panel shows the highest surface density and a pronounced density drop in the core layer, while the incorporation of hazelnut particles leads to a reduction in surface density peaks and a more uniform density distribution across the panel thickness, especially at higher substitution levels. Since the density of the face layers is responsible for the bending features of the panels, it is clearly seen here that the MOE and MOR decrease with the reduction in the face layers’ density. The 100 DB variant (red curve), made with debarked hazelnut particles, demonstrates the most uniform profile, with a less pronounced core density minimum, suggesting improved homogeneity in the internal structure. A similar trend was observed for nettle panels [76]. The density profile can be influenced by various factors such as pressing parameters and the properties of the alternative raw material itself, as each of them responds differently to the pressing conditions. The 85% of the average density level has been added to the plot. Such a level is recognized in the particleboard industry as a minimum density in the core layer. The density drop below this level is strongly connected to the IB, which can be too low in that case. When comparing the density profiles to the IB values, it can be concluded that the variants that have the minimum density in the core zone, close to the mentioned 85% of the average density, represent the decreased IB values.

## 4. Conclusions

The conducted research demonstrated that hazel wood can be a viable alternative raw material in particleboard production. The results showed that the MOE and MOR decreased with increasing hazel wood content, although most variants still met the requirements for P3 and P5 boards, except for the 100% hazel variant in terms of MOE. The IB initially decreased with the addition of hazel wood but increased significantly at higher substitution levels, with the highest IB recorded for the 100% hazel wood variant, surpassing the reference board. WA and TS tests revealed that higher hazel wood content, especially with bark, negatively affected the water resistance of the boards, particularly after 24 h of immersion, exceeding the EN 312 standard limits for panel types P3–P7 for higher substitution levels. In contrast, screw withdrawal resistance improved with the increasing content of hazel wood, which can be attributed to its higher density compared to pine. Bulk density measurements showed that industrial wood particles had the lowest bulk density due to lower pine wood density, while debarked hazel wood particles exhibited the highest bulk density, confirming the influence of bark on particle density. Density profile analysis revealed a characteristic U-shaped curve for all variants, with hazel wood contributing to a more uniform density distribution across the panel thickness, particularly in the 100% debarked hazel wood variant, suggesting improved internal homogeneity. Overall, the results indicate that hazel wood, especially in debarked form and in moderate amounts, can effectively replace industrial wood particles in particleboard manufacturing, though attention must be paid to water resistance properties at higher substitution levels. Regarding the European standard requirements for particleboards, and taking into account the target of maximization of alternative raw material utilization, it can be concluded that the optimum hazel wood particle mass share is about 50%.

Future research could focus on improving the dimensional stability and moisture resistance of hazel wood-based particleboards through treatments such as thermal or chemical modification of hazel particles, surface coating of finished panels, or the incorporation of hydrophobic additives. Additionally, adhesive formulation adjustments, including the use of resin blends or additives to enhance water resistance, could further improve panel performance, particularly at higher hazel wood substitution levels (over 50%).

Based on the findings of this study, hazel wood, especially in debarked form and used in moderate proportions, can be recommended for industrial-scale particleboard production as a sustainable raw material option, provided that measures are implemented to address moisture-related performance issues.

## Figures and Tables

**Table 1:** The configuration of the tested panels.

Panel Name/Variant	Reference/Alternative Raw Material Content [% *w*/*w*]	Density [kg m^−3^]	Thickness [mm]	Resination [%]
0%/reference	100/0	700	13	15
5%	95/5			
10%	90/10			
25%	75/25			
50%	50/50			
100%	0/100			
100 DB ^1^	0/100			

^1^ The hazel wood chips have been debarked before particle production.

## Data Availability

The original data presented in the study are openly available in RepOD at https://doi.org/10.18150/PT8BPA (created and accessed on 10 July 2025).
